# The viscoelasticity of high concentration monoclonal antibodies using particle tracking microrheology

**DOI:** 10.1063/5.0201626

**Published:** 2024-04-24

**Authors:** Conor M. Lewis, Charles T. Heise, Natalia Harasimiuk, Jennifer Tovey, Jian R. Lu, Thomas A. Waigh

**Affiliations:** 1 Biological Physics, Department of Physics and Astronomy, University of Manchester, Manchester M13 9PL, United Kingdom; 2 FUJIFILM Diosynth Biotechnolgies, Billingham TS23 1LH, United Kingdom; 3 Photon Science Institute, University of Manchester, Alan Turing Building, Oxford Rd., Manchester M13 9PY, United Kingdom

## Abstract

The viscoelasticity of monoclonal antibodies (mAbs) is important during their production, formulation, and drug delivery. High concentration mAbs can provide higher efficacy therapeutics (e.g., during immunotherapy) and improved efficiency during their production (economy of scale during processing). Two humanized mAbs were studied (mAb-1 and mAb-2) with differing isoelectric points. Using high speed particle tracking microrheology, we demonstrated that the mAb solutions have significant viscoelasticities above concentrations of 40 mg/ml. Power law viscoelasticity was observed over the range of time scales ( $10^{-4}$–1 s) probed for the high concentration mAb suspensions. The terminal viscosity demonstrated an exponential dependence on mAb concentration (a modified Mooney relationship) as expected for charged stabilized Brownian colloids. Gelation of the mAbs was explored by lowering the pH of the buffer and a power law scaling of the gelation transition was observed, i.e., the exponent of the anomalous diffusion of the probe particles scaled inversely with the gelation time.

## I. INTRODUCTION

Monoclonal antibodies (mAbs) are recombinant proteins with extensive medical applications. There is an ever-increasing demand in the bio-pharmaceutical world for mAbs due to their applications in immunotherapies (e.g., cancer treatment), diagnostics (e.g., pregnancy kits or COVID testing), and cellular labeling.^1^

The formulation of high concentrations of mAb solutions has recently become more important. During immunotherapy (e.g., for the treatment of cancers), mAbs are formulated at increasingly high concentrations to increase dosages (tumours are then less able to evolve resistance) and reduce the number of injections the patient requires. Such mAb formulations often need to be home administered using subcutaneous injections where solution viscosity must be kept <0.05 Pa s to avoid syringe jamming.^2,3^ Another driver for high concentration mAb formulations are new downstream processing techniques in which high concentration solutions can be created with lower costs (due to economy of scale). Thus, mAb solutions prepared at increasingly high dosages (>100 mg ml^−1^)^4,5^ need to be considered.

In general, electrostatically stabilized Brownian colloidal solutions demonstrate significant viscoelasticity as their concentration is increased.^6^ Thus, substantial viscoelasticity is expected for high concentration solutions of mAbs, but has not been observed previously in the literature, presumably due to the extremely high costs of production of mAbs (1 g of mAb can cost upward of £5000) and challenges exist in measuring the viscoelasticity of small volume samples at short time scales. Thanks to the materials supplied by FUJIFILM Diosynth Biotechnologies, the extremely high costs of mAbs were circumvented and we were able to explore the viscoelasticity of microlitres of specimen using particle tracking microrheology at very high frame rates.

The rheology of high concentration mAbs is, thus, little explored due to the large costs involved and challenges in predicting the viscoelasticity of complex colloids. Simple colloidal models can fail to accurately predict mAb viscoelasticity due to complex protein–protein interactions and the consequential folding kinetics.^7,8^

Irreversible aggregation of mAbs through non-covalent interactions between the proteins can give rise to their loss of function and wastage during downstream processing. Such aggregation can be induced by a range of unfavorable conditions, including pH, shear rate, ionic strength, temperature, and surface interactions. Such aggregation of mAbs will give rise to dramatic changes in the viscoelasticity of their suspensions. For example, a gelation phase transition can occur in the extreme limit when high concentrations of unfolded proteins aggregate and percolate the sample volume.^9,10^

The viscoelasticity of mAbs was measured using particle tracking microrheology (PTM) with a fast camera (accessing a range of delay times from 0.1 ms–1 s) and a high resolution optical microscope which allowed small sample volumes (20 *μ*l) to be explored. The linear viscoelasticity was calculated by tracking the thermally driven motion of probe particles^11,12^ using movies from the optical microscope and then calculating their viscoelasticities via the generalized fluctuation–dissipation theorem. The phase behavior of the probe particles needed to be carefully controlled, and polystyrene microspheres were sterically stabilized with polyethylene glycol (PEG) to avoid unwanted sphere–protein–sphere aggregation.

We, thus, examined the linear viscoelasticity of humanized mAbs using PTM over a wide range of concentrations and measured signatures of viscoelasticity for the first time. Furthermore, we reduced the pH of high concentration mAb solutions using an acetic acid buffer and observed a gelation phase transition as the mAbs aggregated. Such gelation is important since it will result in the loss of product efficacy (loss of function during immunotherapies), loss of syringeability and presents challenges for efficient processing, e.g., additional mechanical damage would quickly result during processing. Furthermore, the reduction in pH is often used to inactivate viral impurities, so it is directly relevant to mAb processing. We are not aware of any previous rheological studies of mAb gelation in the literature.

## II. RESULTS AND DISCUSSION

### A. mAb viscoelasticity

Both mAbs were concentrated from initial stock solutions through centrifugal filtration in roughly 50 mg ml^−1^ intervals, taking up to 55 min to reach the densest concentrations. MSDs were constructed as stated previously. Combining a range of videos of tracer particle motion in the solutions, with varying shutter speeds, led to a time delay range of 50 μs–10 s.

**FIG. 1.**
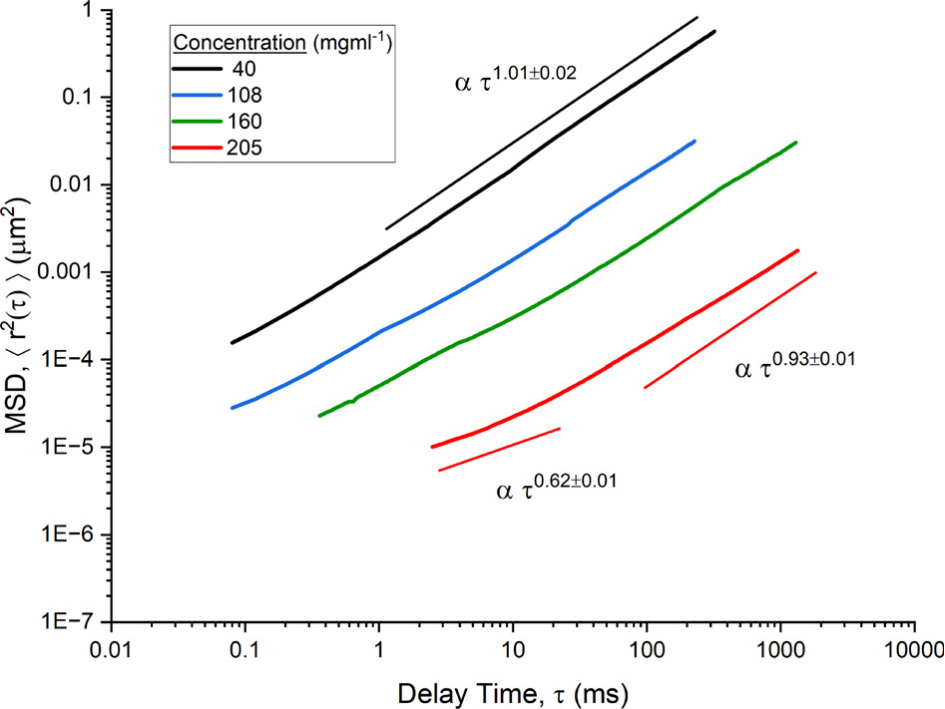
Mean square displacement of PEGylated probe spheres [ $<\Delta r^{2}(\tau )>$] moving in mAb-1 solutions (40–205 mg ml^−1^) as a function of the delay time ( τ) in milliseconds. Trend lines for power law scaling on the time interval are shown.

**FIG. 2.**
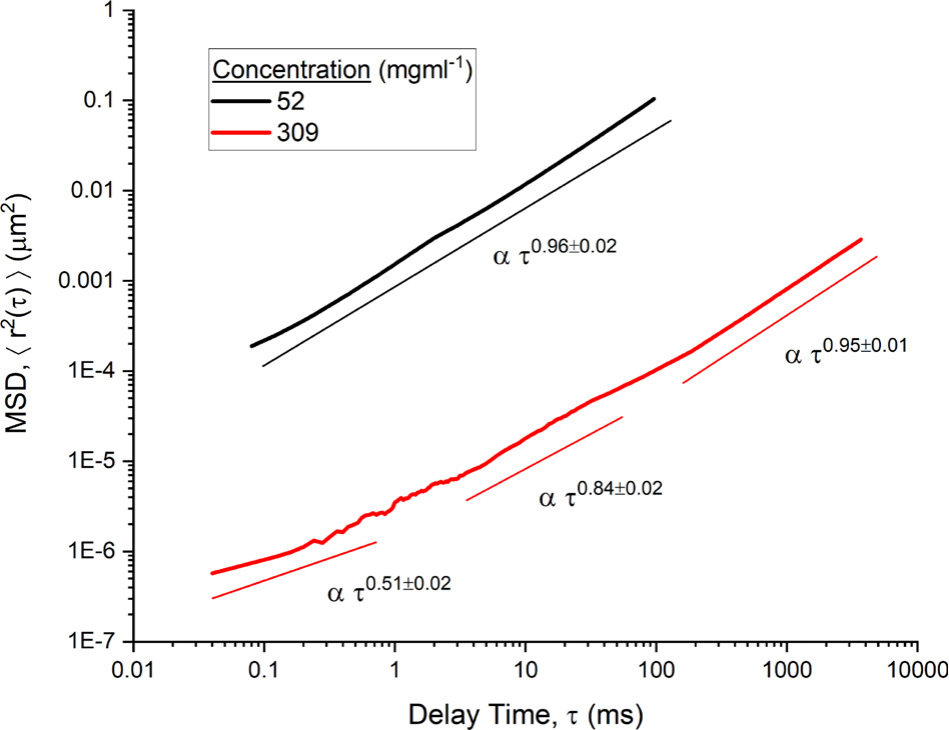
Mean square displacement of PEGylated probe spheres [ $<\Delta r^{2}(\tau )>$] moving in mAb-2 solutions (50 and 309 mg ml^−1^) as a function of the time interval ( τ) in milliseconds. Trend lines for power law scaling on the time interval are shown.

The MSD data of four concentrations of mAb-1 are shown in Fig. 1 and two concentration of mAb-2 are shown in Fig. 2. Sub-diffusive behavior of tracer particles was observed in all but the lowest concentration of mAb-1. In both Figs. 1 and 2, trend lines are used to identify power law relations over specific regions of the entire range of τ. The figures demonstrate a clear decrease in the power law exponent with decreasing delay time. The viscoelasticity of the sample was more marked at higher frequencies (and consequently smaller length scales). This was particularly prominent at higher concentrations where at its lowest, α=0.62±0.01 from 4 to 50 ms for 205 mg ml^−1^ of mAb-1 and α=0.51±0.02 from 0.05–1 ms for 309 mg ml^−1^ of mAb-2, with α tending toward 1 with increasing delay time (the terminal relaxation regime for the fluids). The concentration and lag time dependence of α were indicative of the complex dynamics of antibody interactions. In particular, the viscoelastic nature of antibodies can be attributed to self-association as a result of additional specific attractive interactions at higher concentrations.^13,14^ These attractive interactions were reversible in nature, i.e., the viscoelasticity returns to that for dilute colloidal spheres upon dilution, implying the biological efficacy of the mAbs are maintained.

**FIG. 3.**
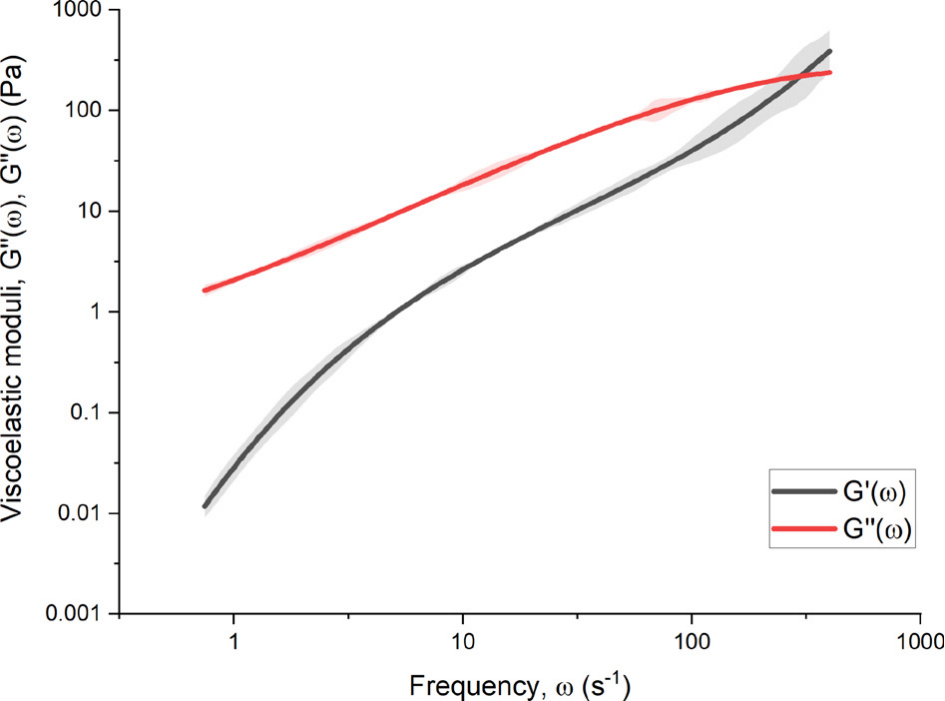
Complex shear moduli [ $G^{\prime }(\omega )$ and $G^{\prime \prime }(\omega )$] as a function of frequency ( ω) for the 205 mg ml^−1^ concentration of mAb-1. Smoothing functions are used to reduce the presence of noise,^15^ represented by the shaded regions.

**FIG. 4.**
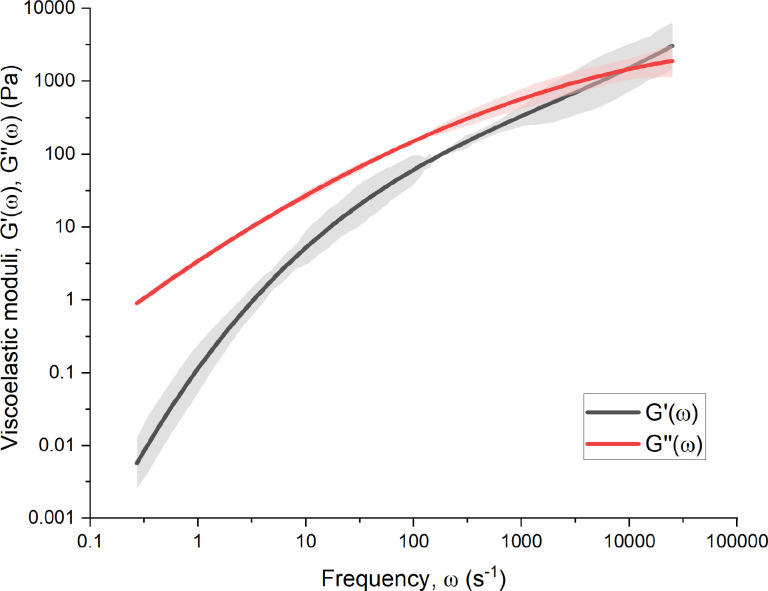
Complex shear moduli [ $G^{\prime }(\omega )$ and $G^{\prime \prime }(\omega )$] are shown as a function of frequency ( ω) for the 314 mg ml^−1^ concentration of mAb-2. Smoothing functions are used to reduce the presence of noise,^15^ represented by the shaded regions.

To understand the separate contributions of viscosity and elasticity to the mAb solutions, graphs of viscoelastic shear moduli [ $G^{\prime }(\omega )$ and $G^{\prime \prime }(\omega )$] as a function of shear frequency ( ω) were constructed for the largest concentrations of both mAbs at 205 and 309 mg ml^−1^ using Eq. (8) (Ref. 15) and are displayed in Figs. 3 and 4, respectively. The crossover frequencies, where $G^{\prime }(\omega )=G^{\prime \prime }(\omega )$ are 297±21 and 8950±320 s^−1^, respectively. The complex shear moduli show that the high concentration mAb solutions are clearly viscoelastic fluids due to the appreciable values of $G^{\prime }$ compared with $G^{\prime \prime }$.

### B. Viscosity-concentration dependence

**FIG. 5.**
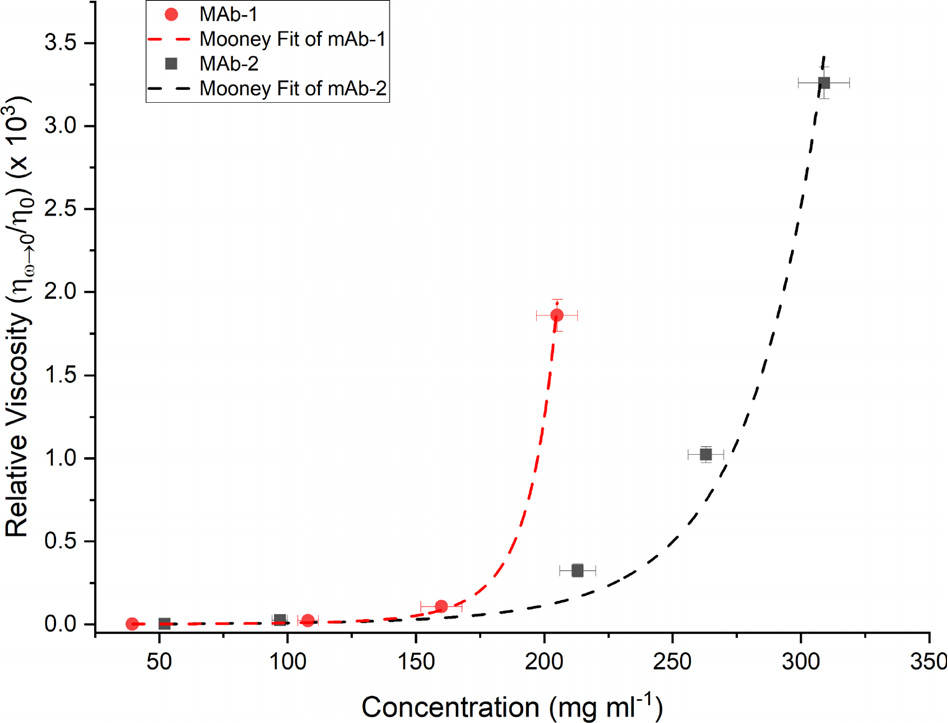
Relative viscosity (low shear solution viscosity over buffer viscosity, $\eta_{R}$) plotted as a function of mAb concentration. A modified Mooney fit was made to both mAb-1 and mAb-2 [Eq. (2)].^18^ Both mAbs see large increases in viscosity with concentrations past 200 mg ml^−1^ up to a factor of 10^3^ above the original buffer viscosity. Error bars account for the inaccuracy in measuring mAb concentrations through mass volume calculations as well as predicting terminal regimes at higher concentrations.

The relative viscosities of the mAb solutions are plotted against mAb concentration in Fig. 5. Solution viscosity ( η) was derived from the terminal regime ( ω→0) of the complex viscosity $\eta^{*}(\omega )$, where

$$\left|\eta^{*}(\omega )\right|=\frac{\left|G^{*}(\omega )\right|}{\omega }.$$ (1)

Many empirical models exist to describe the viscosity of concentrated mAb solutions.^16^ For the mAb solutions investigated, a modified Mooney equation^17,18^ was used, which is a hard quasi-spherical model based on the excluded volume, taking into consideration short-range electrostatic interactions. The relative viscosity ( $\eta_{R}$) can be related to the concentration in the modified Mooney model through

$$\frac{\eta }{\eta_{0}}=\eta_{R}=\text{exp}\left[\frac{[\eta ]c}{1-\left(k/v\right)[\eta ]c}\right],$$ (2)

where $\eta_{0}$ is the buffer viscosity, [η] is the intrinsic viscosity, *c* is the concentration, *k* is a crowding factor, and *v* is a shape determining factor. (k/v) is a dimensionless factor. Relative viscosity increases were observed over factors of 10^3^ for the higher mAb concentrations compared with the buffer viscosity ( $\eta_{0}$). For mAb-1, $[\eta ]=(14.9\pm 3.1)\times 10^{-3}$ ml mg^−1^, (k/v)=0.195±0.095 and for mAb-2 $[\eta ]=(10.0\pm 4.2)\times 10^{-3}$ ml mg^−1^ and (k/v)=0.0391±0.0170. While the curve fits mAb-1 data reasonably well, the mAb-2 fit has a negligibly small value of (k/v).

The modified Mooney model is not expected to account for the long-range electrostatic interactions^19^ that occur with mAb association at larger concentrations. Reversible folding and native aggregation also occur with high concentration antibodies, which will modify the concentration dependence of the relative viscosity. However, the modified Mooney equation provides a reasonable first approximation for the concentration dependence of the current datasets from mAb-1 and mAb-2.

### C. Acid gelation of aggregated mAbs

**FIG. 6.**
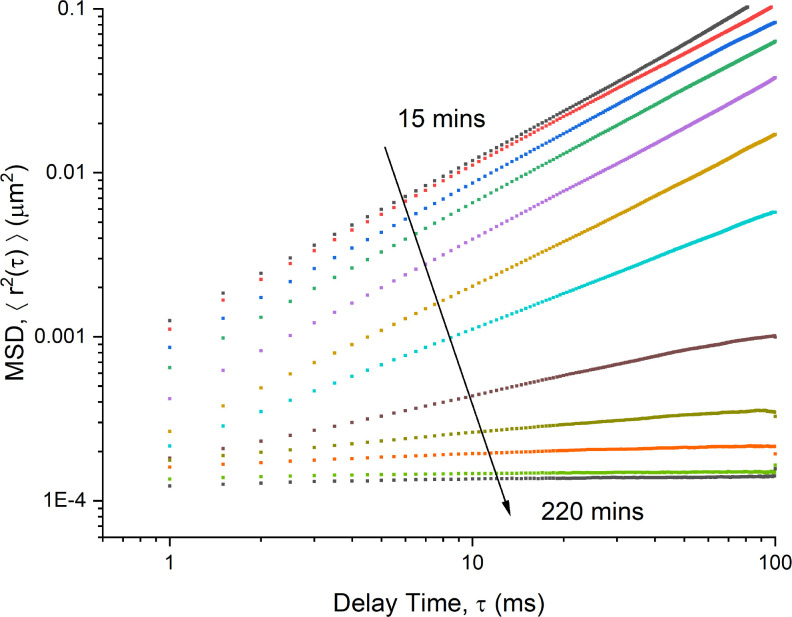
MSDs as a function of delay time for 36 mg ml^−1^ mAb-2 at set times after mixing with 0.4 M of acetic acid. The gelation process spanned over 3 h, with the final measurement at t=220 min. The scaling exponents of each MSD curve are displayed in Fig. 7.

**FIG. 7.**
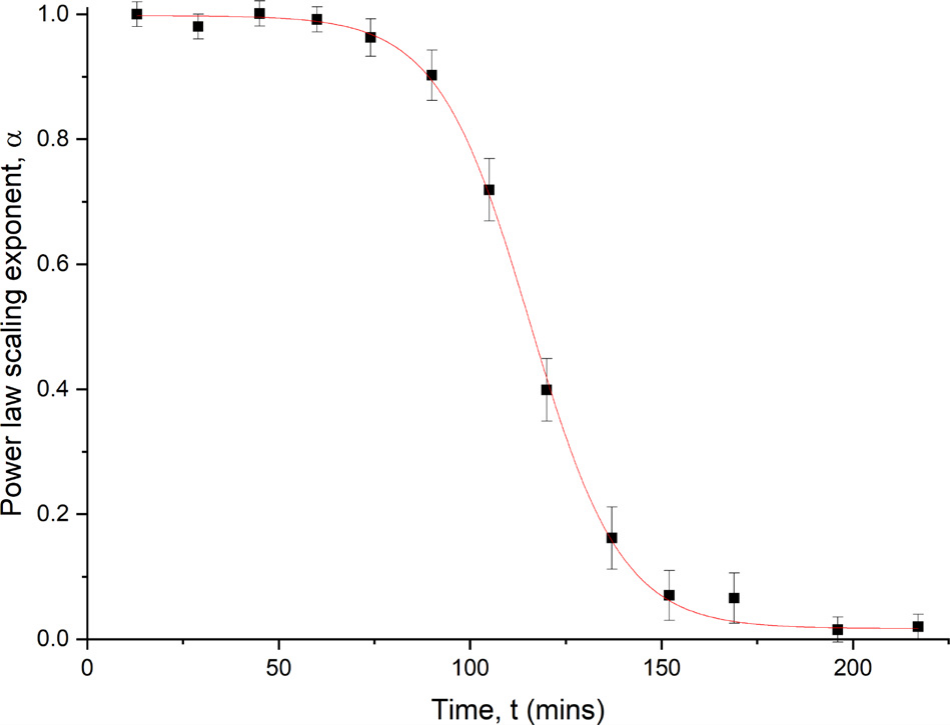
The power law exponent ( α) of the MSDs in Fig. 7 as a function of the time, *t*, after the addition of acetic acid to the solution. An empirical sigmoidal fit function [Eq. (8)] with an intercept of $\alpha_{\text{max}}=0.998\pm 0.001$ and inflection point of $t_{i}=115\pm 0.7$ min provided a good fit. Error bars are given from the root mean-squared difference of additional measurements taken around the data point.

Following the formation of an acidic antibody solution by the addition of acetic acid, MSD data were taken from particle tracking microrheology measurements at specific time intervals. Due to the time sensitive nature of the experiment, a single acquisition time and a camera frame rate were used for all measurements, rather than combining different acquisition times, giving an effective time delay range of 1–100 ms. Figure 6 shows multiple MSDs over a 4 h period. Unlike that observed previously at high concentrations, MSD data followed a single power law expression over the entirety of the measured time intervals, τ,

$$\langle \Delta r(\tau )\rangle \propto \tau^{\alpha },$$ (3)

where α is the power law scaling exponent. α is plotted against the time interval *t* in Fig. 7. An empirical sigmoidal function^20^ was fit to the data

$$\alpha =\alpha_{\text{max}}-\frac{\alpha_{\text{max}}-\alpha_{\text{min}}}{1+\text{exp}[-k(t-t_{i})]},$$ (4)

where $\alpha_{\text{max}}$ is the power law scaling exponent of the antibody solution before the experiment, $\alpha_{\text{min}}$ is the power law scaling exponent by the end of the experiment, *t* is the time after mixing, $t_{i}$ is the inflection point, and *k* is a kinetic gelation constant with units of h^−1^. Limited gelation occurred over the first hour, with the power law scaling exponent ( α) decreasing to approximately 0.9. For the bio-pharmaceutical industry, where both the viral inactivation step of mAbs and column elution during downstream processing involve decreasing the pH of moderately concentrated mAb solutions, mAb-2 demonstrates a reasonable resilience to irreversible aggregation over typical time scales for these experiments.

A gelated load bearing antibody solution was observed after 3 h (lower concentrations of acetic acid increased the gelation time to 48 h) with α exponents approaching values of 0.1. Using the sigmoidal scaling of α as a function of time as a guide, the gelation time can be estimated between 110 and 120 min, where α ranges from 0.6 to 0.4. A gelled solution was observed with dramatically different rheological properties to that seen in the high concentration mAb suspensions, in which the low values of α imply they were viscoelastic power law gels. Gelled samples were load bearing and samples maintained their shape when the cuvettes were inverted.

Much more future work is possible with the microrheology of high concentration mAbs. It would be interesting to explore quantitative colloidal models for the linear rheology of the ungelled suspensions (Figs. 3 and 4). The power law scaling during gelation of mAbs looks very similar to that of small peptide gelation,^21,22^ i.e., a continuous gelation phase transition occurs with universal scaling properties. Optical coherence tomography is a promising technique to explore the flow behavior of antibodies during processing^23,24^ and is robust to the issues with turbid suspensions observed in dynamic light scattering microrheology. Non-linear microrheology, where the sample is actively disturbed through applied forces to a colloidal probe, has not yet been performed on mAbs, but could provide more rheological information on shear thinning effects with gelled samples.^25^ Microrheology could be more extensively used for formulating high concentration mAb solutions for drug delivery, e.g., to understand issues with salting-in and salting-out. Studies of mAb viscoelasticity could provide new insights into optimal processing conditions, e.g., the activity of chromatography columns in which the mAb concentration can be very high (>100 mg/ml).

## III. CONCLUSION

We demonstrated that high concentration mAb solutions have significant elasticity using particle tracking microrheology, likely a result of increased self-association between antibodies. The modified Mooney model provides a reasonable approximation for the solution viscosity as a function of mAb concentration but fails to account for long-range charge interactions and changes of mAb conformations (they are often fairly flexible molecules). We also observe gelation in monoclonal antibodies in acidic (non-native aggregation) conditions and similar phenomena are observed to small peptide and synthetic polymer gelation, i.e., power law viscoelasticity results resembling a continuous phase transition. Viscoelastic behavior in high concentration antibodies caused by self-association, or native/reversible aggregation/folding, affects the rheological properties differently to that in irreversible, non-native aggregation observed during gelation. This is of particular interest for the bio-pharmaceutical industry, since maximum concentrations for syringeability can be determined using particle tracking microrheology, as well as potential problems caused by viral inactivation steps in downstream processing (low pH conditions) observed in the gelation of monoclonal antibodies. Chromatography columns typically function at high mAb concentrations and high viscoelasticity will complicate their optimal functioning, e.g., their kinetics.

## IV. METHODS

Initially, dynamic light scattering (DLS) microrheology was investigated using un-PEGylated silica microspheres.^26^ A Malvern Zetasizer was used. 633 nm laser light was scattered off the immersed probe microspheres with varying mAb concentrations from 1 to 40 mg ml^−1^. Using the intensity autocorrelation function, fluctuations in the speckle of the scattered light could be related to the viscosity of the fluid using the Stokes–Einstein equation,

$$D=\frac{k_{b}T}{6\pi \eta a},$$ (5)

where *D* is the diffusion coefficient of the microspheres measured from the autocorrelaion function, η is the viscosity of the mAb solution, $k_{b}T$ is the thermal energy, and *a* is the radius of the tracer microspheres. Equation (5) is only valid in the regime of a purely viscous fluid at low tracer concentrations. The initial findings showed non-linear power law scaling for the mean squared displacement (MSD) data as a function of time interval indicating power law rheology. Attempting to validate these data with differently sized spheres produced disagreements in the viscosity measurements. There are three likely causes: multiple light scattering from the probe spheres on the interfaces^27^ of the plastic cuvettes, multiple scattering from turbid mAbs, or sphere–protein–sphere interactions causing sub diffusive motion. With DLS, it is difficult to unambiguously diagnose these artifacts. Under a bright field microscope, rapid sphere aggregation was observed, driven by protein–sphere interactions, so the third of the three possible artifacts is clearly contributing. A PEGylation protocol was, thus, required and PEG chains were chemically bonded to the surface of the microspheres to stabilize them. Particle tracking microrheology was then adopted to remove the issues with the other, multiple scattering, artifacts.

### A. Monoclonal antibody structure and formulation

Two humanized mAbs, referenced as mAb-1 and mAb-2, were expressed from Chinese hamster ovary cells and provided by FUJIFILM Diosynth Biotechnologies. Both mAbs are IGG1 class with molecular weights of 72.1 and 71.1 kDa, and iso-electric points of pH 8.17 and 7.37, respectively.

The antibodies were kept at −80°C prior to experiments and gradually thawed to room temperature over 3 h at 19°C. The storage buffers were both sodium phosphate, with mAb-1 in 20 mM sodium phosphate pH 6.0% and 7.5% sucrose, 0.01 % (w/v) polysorbate 20, whilst mAb-2 was in 50 mM sodium phosphate pH 7.5, 7.5% sucrose, 0.01 % (w/v) polysorbate 20.

### B. PEGylation protocol

PEGylation was used to conjugate long chains of polyethylene glycol to the surface of microspheres.^28^ A single step EDC (1-ethyl-3–(3-dimethylaminopropyl)carbodiimide hydrochloride) carbodomide coupling reaction was performed using amine terminated PEG. The addition of a hydrophilic coating on a hydrophobic core can block the nonspecific binding potential of the sphere as well as creating a hydrated shell around each core, limiting aggregation.^29^ 1 μm diameter functionalized polystyrene micro particles (PS-COOH) were purchased from PolySciences with carboxyl surface groups at 25.4 μg^−1^. DLS sizing provided an average hydrodynamic diameter, d = 997 ±7 nm, within the expected variation due to the microsphere polydispersity of 3%. Prior to the experiment, the storage buffer (PBS pH 7.4) of the microspheres was replaced with a 50 mM pH 6.0 sodium phosphate and polysorbate 20 0.01% w/v solution, chosen for its reactive pH with surfactant to maintain sphere stability.

The 10 kDa methoxypolyethylene glycol amine (H_2_NCH_2_CH_2_(OCH_2_CH_2_)_*n*_ OCH_3_) chain was supplied by Sigma–Aldrich, where *n* = 261. This chain length (*n*) is sufficiently large to provide a long-ranged repulsive potential for the chosen probe particle size.^30,31^ The PEG was added at a 1:10 molar excess to the carboxyl group surface concentration to ensure all available surface bonding sites reacted. EDC was mixed with the reagents for 4 h at room temperature. The resultant PEGylated tracer particles were washed in 100 mM Tris to quench any remaining reactive sites and then thrice washed in the appropriate storage buffer. As this was carried out shortly before the microrheology experiments, the storage buffers were that of the respective antibodies detailed in Sec. III.

### C. Particle tracking microrheology

#### 1. Theoretical background

Tracking of multiple monodisperse tracer particles in mAb solutions was used for the microrheology experiments to determine the rheological properties by evaluating the tracers' time averaged mean square displacements (MSD),^32^

$$\mathrm{MSD}=\langle \Delta r^{2}(\tau )\rangle =\langle {\left(r(\tau +t)-r(t)\right)}^{2}\rangle ,$$ (6)

where *r* is the position of the particle, *t* is the time, and τ is the time interval. An ensemble average is also taken over all identified particle tracks in a sample to improve the signal to noise ratio. The MSD is analogous to traditional linear rheometry techniques, where the probe particle exerts a stress on the sample via its thermal motion and the resultant particle displacement in response to this stress is equivalent to the strain experienced by the sample. In purely viscous solutions [i.e., $\langle \Delta r^{2}(\tau )\rangle \propto \tau^{1}$], Eq. (6) can be related directly to the viscosity of the solution through the Stokes–Einstein equation. In sub diffusive conditions [i.e., $\langle \Delta r^{2}(\tau )\rangle \propto \tau^{\alpha }$ where α<1], indicative of both elastic and viscous components in the solutions, the viscosity is dependent on τ and the simple Stokes–Einstein equation does not hold.

To calculate the loss [ $G^{\prime }(\omega )$] and storage [ $G^{\prime \prime }(\omega )$] shear moduli, for the dissipative and elastic components of the solution as a function of frequency ( ω) from the MSD, an altered version of the iRheo software was used,^33^ which is suitable for passive particle tracking microrheology. The compliance, J(τ), is related to the complex viscoelastic shear modulus $G^{*}(\omega )$ through the Fourier transformed compliance $\hat{J}(\omega )$,^34^

$$G^{*}(\omega )=\frac{1}{i\omega \hat{J}(\omega )},$$ (7)

where $J(\tau )=\langle \Delta r^{2}(\tau )\rangle \frac{\pi a}{k_{b}T}$, ω is the frequency, *a* is the radius of the tracer particle, and *kT* is the thermal energy. Through substitution of the second derivatives of the compliance and ensuring the integral is convergent, the complex shear modulus can be explicitly calculated in terms of the experimental data points,^35^

$$\begin{array}{c} \frac{i\omega }{G^{*}(\omega )}=i\omega J(0)+(1-e^{-i\omega \tau_{1}})\frac{\left[J_{1}-J(0)\right]}{\tau_{1}}+\frac{e^{-i\omega \tau_{N}}}{\eta }\\ +\sum_{k=2}^{N}\frac{J_{k}-J_{k-1}}{t_{k}-t_{k-1}}(e^{-i\omega \tau_{k-1}}-e^{-i\omega \tau_{k}}), \end{array}$$ (8)

where J(0) is the intercept at short times, *N* is the total sample points of the data, and $J_{k}$ is the magnitude of the compliance at times $t_{k}$.

#### 2. Experimental setup

PEGylated polystyrene spheres were mixed with mAb solutions just before experiments were conducted at a low 0.01% w/v concentration to avoid rapid aggregation kinetics. Aggregation of the probe microspheres was monitored with the microscope and was negligible for the probes studied. 20 *μ*l of mAb/microsphere solution was placed on a microscope slide within an adhesive spacer, before being sealed with a coverslip. Care was taken to avoid air bubbles, avoiding air–solution interfaces that often cause artifacts in traditional rheometry methods.^36^ A Photron Fastcam camera was attached to a microscope with a bright green LED capable of recording videos from 50 to 10^6^ fps. Due to the relatively turbid samples of high concentration mAbs and the reduced tracer particle numbers with higher frame rates (the field of view was reduced), the maximum frame rate used was 25 000 fps (1024 × 152 pixels^2^) with additional videos taken at 50, 250, and 2000 fps (1024 × 1024 pixels^2^). The tracking accuracy of the data decreases slightly with shorter exposure times due to the reduced number of photons.^32^ By combining multiple videos, a wide range of time scales from 50 μs–10 s were observed.

**FIG. 8.**
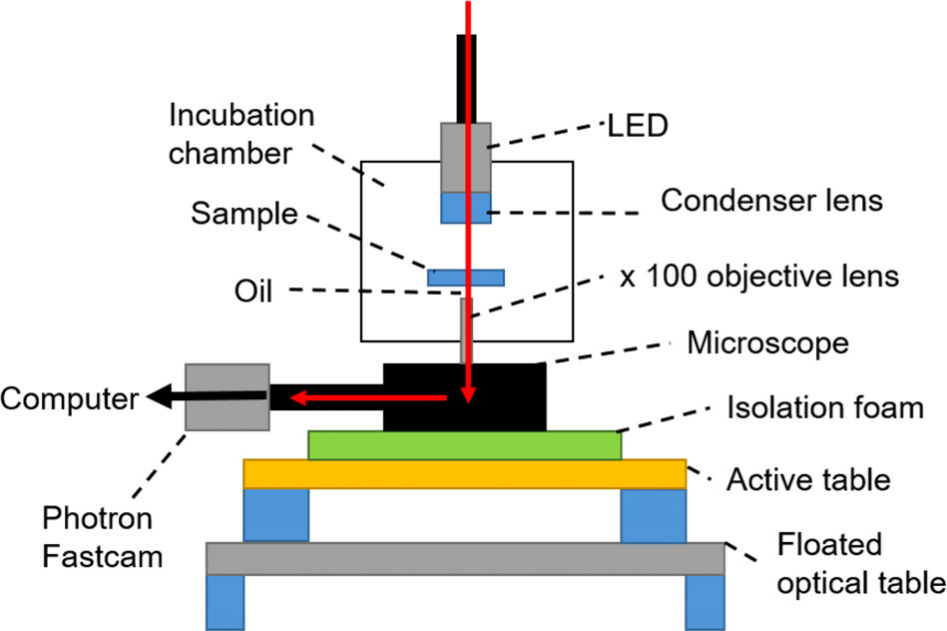
Schematic diagram of the particle tracking microrheology apparatus.^37^ Tracer particles are added to a mAb solution within an environmental chamber to control the temperature. Movies of microsphere motion are taken at varying frame-rates. The active table, isolation foam, and floated optical table reduce the ambient vibrational noise.

The apparatus was enclosed in an incubator to maintain a constant temperature of 25±1°C, with the error due to having to turn off the radiator during measurements on an account of the ambient vibrational noise. The setup is shown in Fig. 8.

Videos were analyzed in PolyParticleTracker,^38,39^ an algorithm designed to track multiple particles between images based on a Gaussian distribution of light around each feature. The tracks were then combined to form the ensemble- and time-averaged MSDs.

Static error in particle tracking microrheology is associated with mis-identification of tracer particle centers between image frames and the finite resolution of the microscope. The static error is observed as a time-independent average error in each of the particles' displacements.^40,41^ Thus, small static errors were observed in high viscosity, high shutter speed samples where tracer particles move little and they were subtracted by calculating the linear intercept at low τ. A drift error is attributed to convective particle movement between individual frames.^40^ The drift error was negligible in the current experiments.

### D. High concentration mAbs and induced aggregation

The concentrations of mAb-1 and mAb-2 were increased beyond the initial values of their stock solutions through centrifugal filtration. A Merck Amicon™ Ultra-0.5 30 kDa molecular weight cutoff filter was used. Antibodies were spun in the device at 14 000 g for varying times corresponding to the final levels of concentration. The final concentrations were calculated using ultraviolet–visible spectroscopy. In cases where the final solution was too viscous to accurately pipette, final concentrations were deduced by dilution and validated with UV measurements of the filtrate giving the appropriate error bars.

Non-native aggregation was induced by mixing a medium concentrated antibody solution (sufficiently concentrated to provide load bearing percolation during gelation) with acetic acid, causing conditions detrimental to the stability of the protein. 1 ml of 52 mg ml^−1^ mAb-2 in storage buffers was mixed with acetic acid giving final solutions of 0.4 M acetic acid, 36 mg ml^−1^ mAb-2. The solution was incubated at 25±1°C for the entirety of the gelation experiment. Tracers particles were added to a small volume of the solution on the first day. An additional control solution was made to ensure that the tracer particles were not affecting the gelation of the solution. Similarly, an attempt was made to mix tracer particles and a gelated antibody solution, but due to the solid-like characteristics of the solution effective mixing of the tracer particles proved impossible.

## Data Availability

The data that support the findings of this study are available from the corresponding author upon reasonable request.
